# Semi-supervised skin cancer diagnosis based on self-feedback threshold focal learning

**DOI:** 10.1007/s12672-024-01043-8

**Published:** 2024-05-22

**Authors:** Weicheng Yuan, Zeyu Du, Shuo Han

**Affiliations:** 1 College of Basic Medicine, Hebei Medical University, Zhongshan East, Shijiazhuang, 050017 Hebei China; 2 Department of Anatomy, Hebei Medical University, Zhongshan East, Shijiazhuang, 050017 Hebei China; 3 School of Health Science, University of Manchester, Sackville Street, Manchester, 610101 England UK

**Keywords:** Skin cancer diagnosis, Semi-supervised learning, Self-feedback threshold learning, Focal learning

## Abstract

Worldwide, skin cancer prevalence necessitates accurate diagnosis to alleviate public health burdens. Although the application of artificial intelligence in image analysis and pattern recognition has improved the accuracy and efficiency of early skin cancer diagnosis, existing supervised learning methods are limited due to their reliance on a large amount of labeled data. To overcome the limitations of data labeling and enhance the performance of diagnostic models, this study proposes a semi-supervised skin cancer diagnostic model based on Self-feedback Threshold Focal Learning (STFL), capable of utilizing partial labeled and a large scale of unlabeled medical images for training models in unseen scenarios. The proposed model dynamically adjusts the selection threshold of unlabeled samples during training, effectively filtering reliable unlabeled samples and using focal learning to mitigate the impact of class imbalance in further training. The study is experimentally validated on the HAM10000 dataset, which includes images of various types of skin lesions, with experiments conducted across different scales of labeled samples. With just 500 annotated samples, the model demonstrates robust performance (0.77 accuracy, 0.6408 Kappa, 0.77 recall, 0.7426 precision, and 0.7462 F1-score), showcasing its efficiency with limited labeled data. Further, comprehensive testing validates the semi-supervised model’s significant advancements in diagnostic accuracy and efficiency, underscoring the value of integrating unlabeled data. This model offers a new perspective on medical image processing and contributes robust scientific support for the early diagnosis and treatment of skin cancer.

## Introduction

Skin cancer ranks among the most common types of cancer globally, with its etiology involving exposure to ultraviolet radiation, genetic predispositions, and environmental factors. Increased ultraviolet exposure, due to rising global temperatures and more outdoor activities, correlates strongly with the growing incidence of skin cancer, highlighting its public health significance [1–3]. Additionally, genetic and environmental elements play pivotal roles in the pathogenesis of skin cancer. With societal progression and environmental alterations, the impacts of these causative elements have become increasingly pronounced, positioning skin cancer as an unignorable public health challenge. In regions with high exposure to ultraviolet radiation, the incidence and mortality rates of skin cancer are particularly alarming. This trend strains healthcare systems and exacts considerable economic and emotional tolls on patients and families [4, 5]. Furthermore, the escalating costs of skin cancer treatment have made it challenging for some patients to access timely and effective care.

Timely and effective diagnosis and treatment of skin cancer are vital to mitigate the health burden it imposes. Early diagnosis not only enhances treatment outcomes but also considerably reduces treatment costs. Early-stage skin cancer’s subtle symptoms often resemble those of other skin conditions, especially the highly malignant melanoma, significantly increasing mortality risk if treatment is postponed [6, 7]. Despite skin cancer diagnosis relying on clinical evaluation and biopsies, its similarity to common skin diseases heightens the risk of misdiagnosis, especially by non-specialists, presenting a notable diagnostic challenge.

Furthermore, with the advancement of medical technology, especially the application of artificial intelligence (AI) in medicine [8–10], new possibilities for the diagnosis and treatment of skin cancer have emerged. The use of AI for image analysis and pattern recognition can assist doctors in enhancing the precision and efficiency of diagnosis, especially playing a crucial role in the diagnosis of early-stage skin cancer. Therefore, integrating traditional medical knowledge with advanced deep learning technologies carries significant practical implications for the effective diagnosis and treatment of skin cancer.

Several challenges are faced in the AI-based diagnosis of skin cancer, among which the most crucial is the effective processing and analysis of a large amount of medical imaging data. Currently, the automated diagnosis of skin cancer heavily relies on AI technology, particularly methods based on supervised deep learning [11, 12]. The essence of this approach is to utilize a vast number of labeled medical images to train the algorithm to recognize and categorize skin cancer. While this method has achieved success to some extent, it also faces some significant limitations. Firstly, the process of labeling medical images is not only time-consuming and labor but also costly. Additionally, the scarcity of high-quality labeled images limits training data diversity, affecting the accuracy and generalizability of supervised models. This situation directly affects the generalizability and diagnostic accuracy of supervised learning methods. In actual clinical environments, a large number of unlabeled medical images are unused, failing to be fully utilized, which further restricts the development of skin cancer diagnostic technology.

In real-world application scenarios, many supervised learning models trained on specific datasets prove effective only within the context of their original training environments. A significant hurdle emerges when adapting these models to different scenarios; it entails the reannotation of a vast of data and retraining of the model for compatibility with the new context. Additionally, a prevalent issue in such application scenarios is the imbalance in the number of patients across different categories, which profoundly impacts both the training and the efficacy of the model. This imbalanced data distribution often results in models that are biased towards the majority class, thereby diminishing their diagnostic accuracy for less represented classes.

This situation underscores the crucial research problem addressed in this study: how to utilize the abundance of unlabeled samples in a new domain, with minimal labeling cost, to jointly learn a robust diagnostic model for skin cancer that is also sensitive to the issue of class imbalance. This is particularly pertinent as the ability to generalize across diverse clinical settings without extensive fully-annotating, and in a manner that accurately represents all patient categories, performing a significant advancement in the field.

**Fig. 1:**
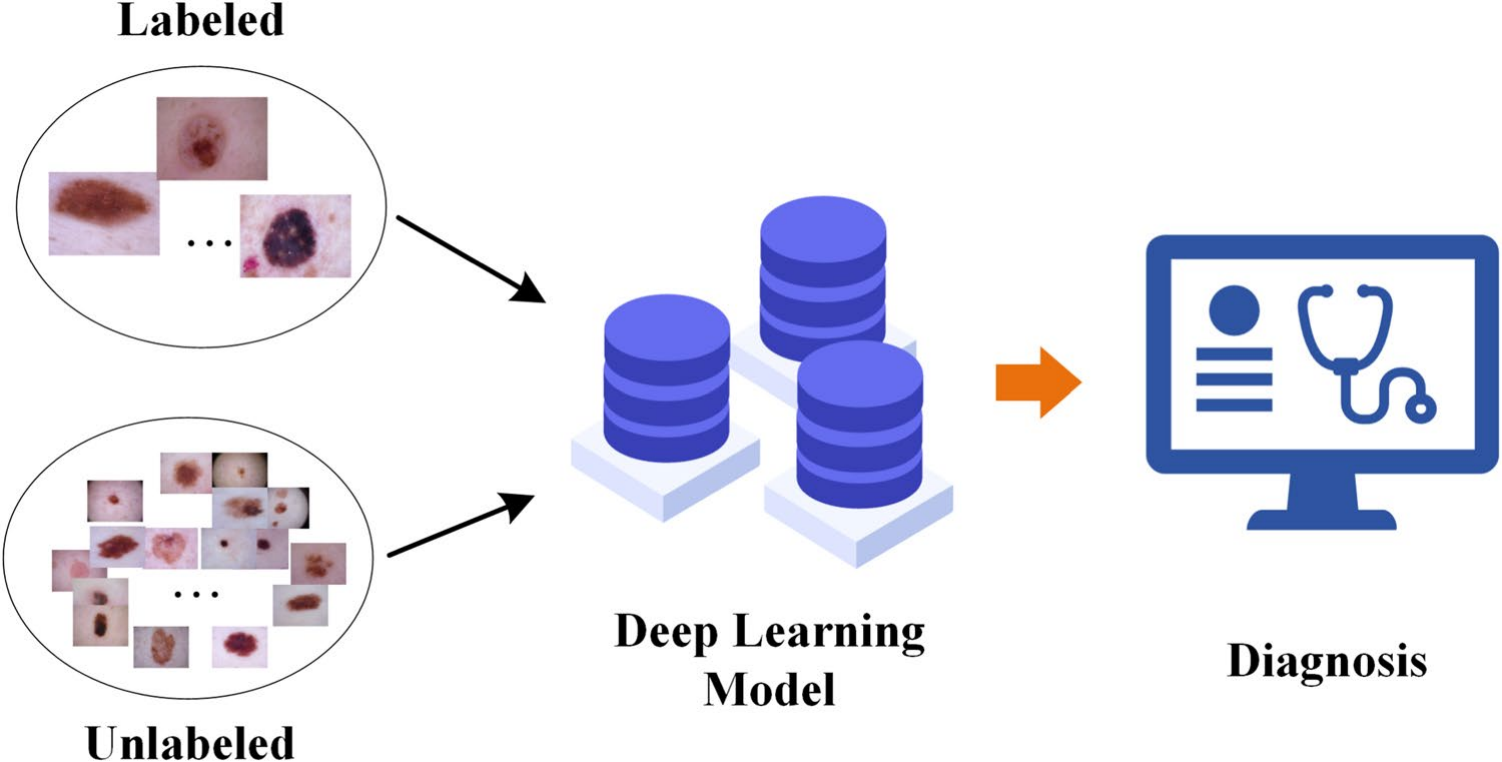
Schematic diagram of a semi-supervised skin cancer diagnosis model

In light of these challenges, we propose an innovative semi-supervised method for diagnosing skin cancer. The crux of this method lies in its utilization of not only limited labeled data but also a vast amount of unlabeled data for training the deep learning classification models, as shown in Fig. 1. Within this framework, we introduce a Self-feedback Threshold Focal Learning (STFL) method, an innovative technique aimed at enhancing the model’s performance when dealing with unlabeled data.

The key to the STFL model lies in its ability to dynamically adjust the selection threshold for unlabeled samples during the model training process. This implies that the model can self-regulate based on the distribution and characteristics of existing data, thereby more accurately distinguishing between benign and malignant tumor tissues in unlabeled data, and selecting reliable unlabeled samples for further training the diagnostic model. This method significantly reduces the misdiagnosis rate on unlabeled data, while simultaneously enhancing the model’s discriminatory capacity and diagnostic accuracy. This model leverages existing imaging resources more efficiently, markedly improving diagnosis accuracy and process efficiency. It overcomes traditional supervised learning’s limitations and augments skin cancer diagnostic capabilities, promising enhanced treatment outcomes and patient quality of life.

### Motivations

The major motivations from clinical scenario and model applications are concluded as follows,

- *Ubiquity and Severity on a Global Scale* Skin cancer is one of the most common types of cancer worldwide, and timely and effective diagnosis is crucial for alleviating public health burdens.
- *Limitations of Existing Methods* Traditional supervised learning approaches have achieved some success in skin cancer diagnosis, but due to their reliance on extensive labeled data, these methods face limitations and challenges associated with data annotation.
- *The Potential of Unlabeled Data Utilization* There is an abundance of unlabeled medical image data in actual clinical settings that remain underutilized, presenting an opportunity to enhance the accuracy and efficiency of skin cancer diagnostic techniques.
- * Advancements in Artificial Intelligence Technologies* With the progress of medical technology, particularly the application of artificial intelligence in medical diagnostics, it has become possible to propose new diagnostic methods to improve the precision and efficiency of early skin cancer diagnosis.

### Contributions

Aiming at solving the aforementioned challenges, our proposed STFL approach achieves following contributions.

- *Development of a Novel Semi-Supervised Learning Method* Our study introduces a Semi-Supervised Skin Cancer Diagnosis model based on Self-Feedback Threshold Focal Learning (STFL), which utilized ResNet-50 as the feature extractor to support the semi-supervised learning, which effectively save annotating cost and solving the class-imbalance problem in skin cancer diagnostic model training.
- *Dynamic Adjustment of Selection Threshold for Unlabeled Samples* The STFL model dynamically adjusts the selection threshold for unlabeled samples during the training process, efficiently filtering reliable unlabeled samples and mitigating the impact of class imbalance through focal learning.
- *Experimental Validation and Improved Diagnostic Accuracy* Experiments conducted on the HAM10000 dataset demonstrate that our model achieves satisfactory accuracy with as few as 500 labeled samples, proving that our proposed model can enhance the accuracy and efficiency of skin cancer diagnosis even with a limited number of labeled samples.
- *New Perspectives and Scientific Support for Early Diagnosis and Treatment* Our model not only overcomes the limitations of traditional supervised learning methods but also provides a fresh perspective for medical image processing, offering robust scientific support for the early diagnosis and treatment of skin cancer.

To substantiate the efficacy of our proposed solution, this paper simulates real-world scenarios on a fully annotated public dataset by training the model using a combination of partially labeled images and a substantial volume of unlabeled data under assumption, while also addressing class imbalance. During the model testing phase, we employ a comparative verification process that utilizes existing data labels against the model’s predictive outputs, thereby facilitating a quantitative evaluation of the model’s performance, including its ability to accurately diagnose across various patient categories. This approach not only underscores the practicality of our method in addressing the outlined challenges but also affirms the proposed semi-supervised learning model’s capacity to enhance the accuracy and efficiency of skin cancer diagnosis within varied and unstandardized clinical environments, even amid class imbalance. Through this validation, we aim to demonstrate the potential and versatility of our model, offering new perspectives in transferring the semi-supervised learning in new scenarios, significantly contributing to the practical early diagnosis and effective treatment of skin cancer by ensuring equitable diagnostic accuracy across all patient categories.

The organization of this paper is meticulously crafted into five sections, starting with an **Introduction** that establishes the need for advanced AI in early skin cancer diagnosis and details the limitations of traditional methods. **Related Works** situates the study within the broader research context, identifying the space that our STFL model intends to fill. The **Method** section delves into the model’s mechanics and its strategic approach to scarce labeled data and class imbalances. The **Experiments** section demonstrates the model’s effectiveness through results from the HAM10000 dataset, underscoring the model’s performance with limited annotations. This paper culminates in the **Discussion and Conclusion**, which ties together the study’s outcomes, its impact on the field, and proposes avenues for future research, marking our distinct contribution to medical image analysis and skin cancer diagnostic processes.

## Related work

In recent years, notable advancements have been made in the field of automated diagnosis of skin cancer, particularly through the application of deep learning and other intelligent algorithms. These works are divided into machine learning-based, deep learning-based, and semi-supervised learning-based methods.

### Machine learning-based methods

In early stage, skin cancer diagnosis is achieved by traditional learning-based models [13–16], which deploy machine learning models on the specific description features. Saez et al. [13] classified melanoma based on the thickness of the lesion by three values, combined with Logistic Regression (LR) model for classification; Oliveira et al. [14] utilized Support Vector Model (SVM) to classify lesion feature from an isotropic diffusion filter. Noroozi et al. [15] combined two or three Fourier transform features to form one Z-transform feature; After traditional machine learning application, hybrid techniques involving machine learning and deep learning methods are proposed. Such as, Yu et al. [16] presented a joint framework combining deep learning and local description encoding strategy for the melanoma recognition in dermoscopy images.

Traditional machine learning-based methods for skin cancer diagnosis, while pioneering, present inherent limitations, particularly in handling high-dimensional data and the need for extensive feature engineering. These methods require domain expertise to identify and extract relevant features from medical images, which are often time-consuming and may not capture intricate patterns that are vital for accurate diagnosis. Moreover, they generally lack the ability to learn feature representations in an end-to-end manner, resulting in suboptimal performance when dealing with the complex and subtle variations present in dermatological imagery. In contrast, the advent of deep learning, offers a promising alternative for skin cancer diagnosis.

### Deep learning-based methods

Deep learning-based models have not only improved the accuracy of diagnosis but also enhanced the efficiency of the diagnostic process. For instance, Gajera et al. [17] performed a comprehensive analysis among eight pre-trained CNN models for melanoma diagnosis; Pacheco et al. [18] combined images and metadata features using deep learning models applied to skin cancer classification; Abd Elaziz et al. [19] employed MobileNetV3 and the Improved Artificial Rabbit Optimizer, achieving enhanced accuracy through sophisticated feature refinement. Gajera et al. [20] proposed an ensemble CNN feature fusion and sparse autoencoder based framework to improve melanoma classification performance; Sethanan et al. [21] introduced a Skin Cancer Classification System combining image segmentation, CNNs, and a dual Artificial Multiple Intelligent System, resulting in over 99.4% accuracy in skin cancer classification. Bassel et al. [22] developed a stacking classifier method using Resnet50, Xception, and VGG16 that reached 90.9% accuracy for melanoma and benign skin cancer detection. These contributions signify progress toward more accurate and universally applicable skin cancer diagnostic methods. Gajera et al. [23] developed a multi-CNN ensemble approach for multi-class skin lesion classification. These studies demonstrate that the automatic diagnosis of skin cancer is advancing towards higher precision and better generalizability through continuous algorithmic innovations and model structural developments.

However, most existing studies predominantly rely on supervised learning models, which require support from extensive labeled data. In practical applications, this demand often proves difficult to fulfill, as acquiring accurately labeled medical imaging data is typically costly and scarce. Particularly in the field of skin cancer, the shortage of high-quality labeled data becomes a significant barrier to enhancing the accuracy and generalizability of automatic diagnosis systems. Therefore, introducing semi-supervised learning models serves as a critical approach to addressing this issue. Semi-supervised learning effectively leverages sparse labeled data in conjunction with abundant unlabeled data to improve diagnostic accuracy for complex skin conditions. Against this backdrop, the present study proposes a semi-supervised self-feedback threshold focal learning model for skin cancer diagnosis. This model aims to overcome the limitations of existing supervised learning methodologies, providing a more effective and accurate tool for early diagnosis and treatment of skin cancer.

### Semi-supervised learning-based methods

In addressing the challenges posed by the extensive requirement for annotated datasets in skin cancer diagnosis, semi-supervised learning (SSL) approaches have emerged as a promising avenue for reducing the annotation burden while enhancing diagnostic performance in novel clinical scenarios. By exploiting the synergy between a limited pool of labeled data and a larger corpus of unlabeled images, SSL methods offer a strategic balance between manual annotation effort and model accuracy. Among early explorations in this domain, Bdair et al. [24] innovated a semi-supervised federated learning approach that leverages peer learning for categorizing skin lesions, highlighting the potential of decentralized learning in medical image analysis. Meanwhile, Masood et al. [25] introduced a semi-advised training and classification strategy, demonstrating the efficient utilization of scarce labeled resources alongside the vast availability of unlabeled data for skin cancer diagnosis. Further, Agarwal et al. [26] engineered a novel framework utilizing deep convolutional adversarial networks, which autonomously learns feature representations of melanoma samples in a semi-supervised manner. These studies collectively underscore the evolving landscape of semi-supervised learning methodologies in skin cancer diagnosis, marking a critical step toward leveraging unlabeled data to overcome traditional constraints in medical imaging diagnostics.

While previous semi-supervised learning models have made some strides in skin cancer diagnosis by utilizing unlabeled data, these methods have not fully resolved the inherent class imbalance issue. This imbalance can bias models towards overrepresented classes, detracting from the accuracy in detecting rarer, yet often more serious, cancer types. Our Self-feedback Threshold Focal Learning (STFL) addresses this imbalance head-on by dynamically modifying the threshold for including unlabeled samples, aiming at a more equilibrated and sensitive approach that improves the detection of less common cancers. This innovative solution enhances diagnostic accuracy and contributes to the advancement of semi-supervised learning applications in medical diagnostics.

## Our proposed self-feedback threshold focal learning

### Overview of our model

This paper introduces a semi-supervised skin lesion classification model, the STFL (Self-feedback Threshold Focal Learning) network, which adapts threshold learning to fully leverage unlabeled medical imaging data. Firstly, an initial model is trained based on the ResNet network [27, 28] using already labeled skin lesion data. Subsequently, this initial model is employed to predict unlabeled skin images, and pseudo-labels are generated based on these predictions. By using the self-feedback threshold focal learning method, it automatically calculates the global self-feedback threshold and category-specific self-feedback thresholds, selecting highly reliable samples. The primary purpose of this operation is to utilize the substantial amount of unlabeled images and assign them class labels so that they can be integrated into further model training. It should be noted that the pseudo-labels generated through model prediction will inevitably harbour certain degrees of noise and uncertainty. Within our model, the removal of these noisy pseudo-labeled samples is of utmost importance. Simultaneously, class imbalance among the samples after sample selection also becomes a critical factor affecting subsequent training effectiveness. This further trains and optimizes the model with focal learning, fully exploiting the discriminatory information in unlabeled samples, thereby developing a robust skin cancer diagnosis model.

**Fig. 2:**
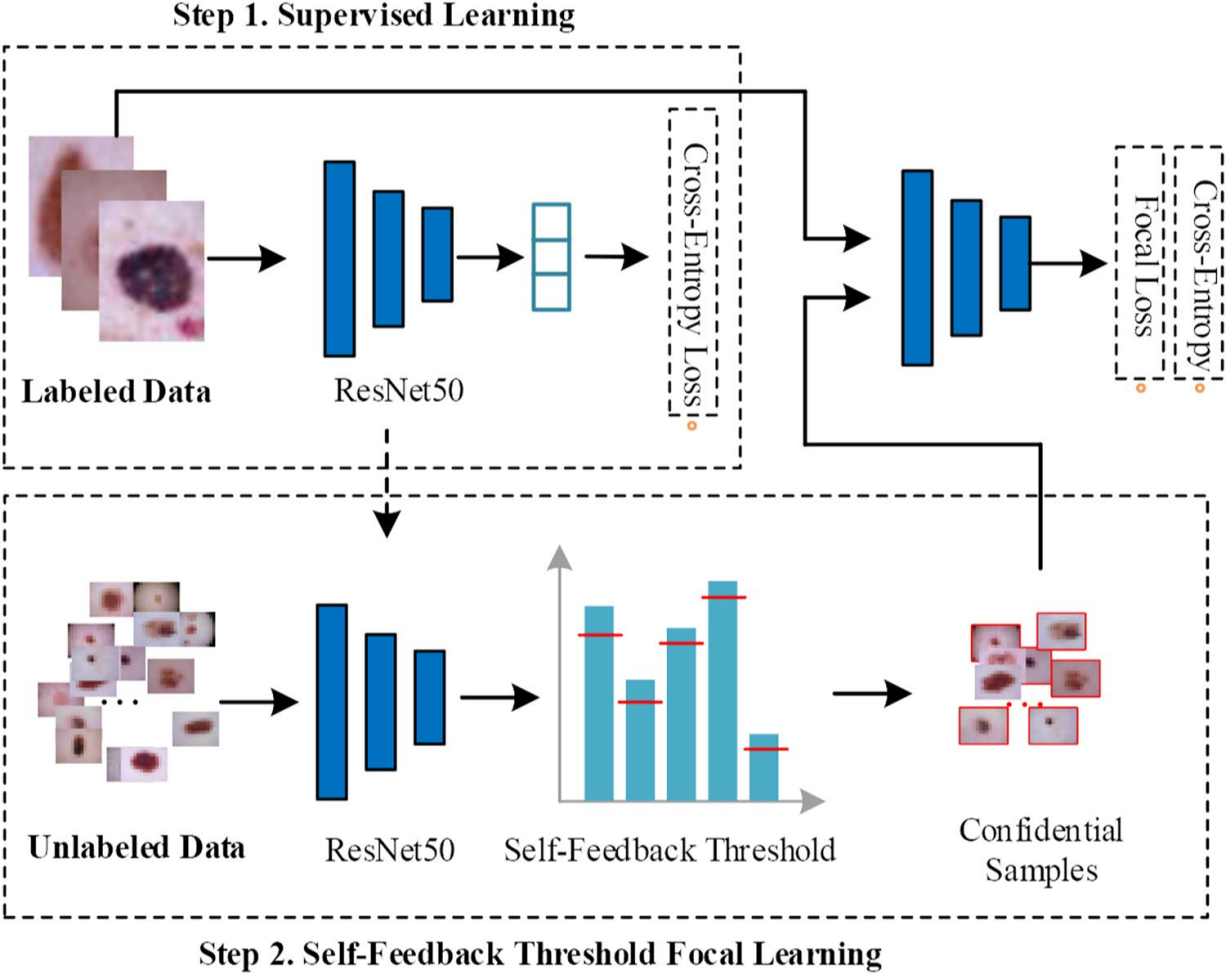
The framework of proposed STFL model. STFL employs labeled data to train a ResNet feature extractor, then applies self-feedback threshold focal learning to select and utilize reliable unlabeled samples, enhancing the model’s robustness in skin cancer diagnosis

Regarding the labeling process for unlabeled samples, a fixed prediction probability threshold is typically set to screen pseudo-labeled samples, ensuring that the samples with higher prediction confidence are included in the subsequent model training process. Existing threshold setting methods are often based on a fixed confidence threshold, which could lead to a severe prediction bias in the case of class imbalance. The STFL module developed in this paper designs a method for automatically calculating the threshold, which replaces the manual threshold setting method, and incorporates an imbalanced class focal learning mechanism. Specifically, it uses a method for selecting unlabeled samples based on self-feedback threshold focal learning, which consists of three parts: global self-feedback threshold learning, intra-class self-feedback threshold learning, and an class imbalance focal learning module. This method refines category-specific confidence thresholds using model predictions to improve reliability and confidence in the input images. It makes full use of the information from unlabeled images, thereby improving the model’s generalizability and performance. Moreover, our model introduces supervised learning loss, unsupervised learning loss, and focal loss for training and optimizing the parameters of the semi-supervised skin cancer diagnosis model. The network architecture of our method is drawn in Fig. 2.

### Problem definition

In the semi-supervised skin cancer diagnosis task, the training data includes both labeled and unlabeled skin image data. In this paper, we define the labeled samples as $X_l=\{x^l_1,x^l _2,\cdots , x^l _{N_l}\}$, where $N_l$ represents the number of labeled samples, and the corresponding lesion category labels are defined as $Y_l=\{y_1,y_2,\cdots ,y_{N_l}\}$. Likewise, the set of unlabeled skin lesion images is defined as $X_u=\{x^u_1,x^u_2,\cdots ,x^u_{N_u}\}$, where $N_u$ represents the number of unlabeled samples. For feature extraction, this paper utilizes ResNet50 as the backbone network $f(\cdot )$ to extract image features, and a classification layer $p(\cdot )$ is defined to predict the feature categories.

### Supervised learning based on labeled skin images

To ensure the capability of the backbone network to extract the features of skin lesion images, this model initially trains the feature extraction abilities of the network based on the labeled samples $X_l$ and their category labels $Y_l$, introducing a cross-entropy loss ($\mathcal {L}_{ce}$). The loss is defined as follows,

1 $$\begin{aligned} {{\mathcal {L}}_{ce}}=-{{\mathbb {E}}_{x_{i}^{l}\in {{X}_{l}}}}{{y}_{i}}\log p(f(x_{i}^{l})) \end{aligned}$$

After an initial training with the above supervised loss function, the feature extractor acquires a certain capability to extract skin lesion features. This can be used to predict the probability distribution of unlabeled samples, thereby implementing the pseudo-label generation process for unlabeled samples. The primary purpose of using labeled samples to train the feature extractor is to assign category labels to unlabeled skin lesion images, thus incorporating them into the training set for subsequent training. By introducing pseudo-labels, we expand the original labeled dataset and include unlabeled samples in the training set, allowing the model to optimize with more data during the training process. Given an unlabeled skin lesion image sample $x^u_i$, one can obtain the predicted score for each possible category $p^u_i=p(f(x^u_i))$. This score represents the model’s confidence that the sample belongs to each category, and the category corresponding to the maximum predicted probability is the predicted label.

### Unlabeled sample selection model based on fixed threshold

In the process of generating pseudo-labels for unlabeled skin lesion images, it is inevitable that some noise samples with incorrect labels will be produced. Therefore, in this model, it becomes crucial to remove these pseudo-labeled samples containing noise.

Upon obtaining the predicted probability $p^u_i$ for each category of the unlabeled skin image data $x^u_i$, pseudo-label generation models generally set a fixed high threshold $T_{fix}$ to filter out reliable unlabeled data based on their prediction results. This is based on the assumption that if the model’s predicted probability exceeds this fixed threshold, it is regarded as a pseudo-label, and the reliable sample selection process is described in the formula. For an unlabeled sample $y^u_i$, if the predicted score of a certain category exceeds the set threshold, the sample will be classified into this category, resulting in $y^u_i$; conversely, if the scores of all categories are below the threshold, the sample might be judged as indeterminable and not used for the next step of model training. In this way, these filtered samples with pseudo-labels can be added to the training set for the next round of model training. Based on this fixed threshold selection strategy for unlabeled samples, the selected reliable samples can be used to further train the model as Eq. 2.

2 $$\begin{aligned} {{\mathcal {L}}_{T}}=-{{\mathbb {E}}_{x_{i}^{u}\in {{X}_{u}}}}\mathbbm {1}(\max (p_{i}^{u}(j))>{{T}_{fix}})\cdot (y_{i}^{u}\log p(f(x_{i}^{u}))) \end{aligned}$$

Here, $p^u_i(j)$ represents the probability of belonging to the *j*-th category in the prediction results, and $\mathbbm {1}$ signifies the indicator function of the sample exceeding the fixed probability threshold. Consequently, in $\mathcal {L}_T$, only the unlabeled samples with prediction probabilities greater than $T_{fix}$ are retained for model training.

Although setting a higher fixed probability threshold can filter out more reliable unlabeled samples for model training, a globally fixed threshold cannot adapt to the probability distribution of each category, leading to considerable differences in sample selection across different categories. This imbalance in category sample selection results in further model training being biased towards categories with more samples.

### Self-feedback adaptive threshold learning

In order to overcome the limitations brought about by the traditional fixed threshold-based methods, this study improves this method and proposes a novel unlabeled sample model selection strategy and class imbalance focal learning mechanism based on self-feedback adaptive threshold learning. This strategy encompasses both global adaptive feedback and category-specific adaptive feedback mechanisms. Influenced by the related literature [29], this study posits that the key to determining the threshold in semi-supervised learning (SSL) lies in the threshold’s ability to reflect the state of learning. By evaluating the prediction confidence of a precisely calibrated model, the learning effect can be effectively gauged. On this basis, the study introduces the Self-feedback Threshold Focal Learning (STFL) method. This method utilizes the model’s predictions during the training process to automatically define and dynamically adjust the confidence threshold for each category. Initially, the STFL method estimates a global threshold as the Exponential Moving Average (EMA) [30] of the model’s confidence. Subsequently, the STFL evaluates the EMA of the model’s probabilities for each category, adjusts the global threshold, and combines it with category-specific local thresholds to reduce the class imbalance in sample selection. In the early stages of training, as the model is not yet fully trained, the threshold is set lower to include more potentially correct samples. As the model’s predictive ability and confidence increase, the threshold is adaptively raised to exclude potentially inaccurate samples, thus mitigating the risk of classification bias.

The STFL primarily comprises global self-feedback threshold learning, intra-class(local) self-feedback threshold learning, and a class-imbalance focal learning module. The global threshold primarily controls the overall sample selection, while the local threshold aims to adjust the global threshold in a category-specific manner, considering intra-class diversity and potential category proximity. Concurrently, the class-imbalance focal learning module targets the class imbalance in the selected sample set for specific model training, reducing the negative impact of class imbalance on model training.

#### Global self-feedback threshold learning

In the STFL method, the setting of the global threshold needs to be closely related to the model’s confidence in the unlabeled data, so as to accurately reflect the overall learning situation. Furthermore, to effectively exclude incorrect pseudo-labels, this global threshold should gradually increase during the training process. In this study, we define the global threshold $T_g(t)$ as the average confidence of the model in the unlabeled data at the *t*-th iteration. The optimization of the global threshold is iteratively updated according to the approach outlined in literature [31].

3 $$\begin{aligned} {{T}_{g}}(t)=\left\{ \begin{array}{*{35}{l}} \frac{1}{{{N}_{c}}}, &{} \text { if }t=0 \\ \lambda {{T}_{g}}(t-1)+(1-\lambda )\frac{1}{B}\sum \limits _{b=1}^{B}{\max }\left( p_{i}^{u}(j) \right) , &{} \text { otherwise } \\ \end{array} \right. \end{aligned}$$

In this equation, $N_c$ represents the number of categories, and $\lambda$ is the parameter for the Exponential Moving Average (EMA).

#### Intra-class self-feedback threshold learning

In the STFL method, the purpose of the intra-class self-feedback threshold is to adjust the global threshold in a category-specific manner, thus accounting for intra-class diversity and potential category proximity. This is achieved by calculating the expected prediction value of the model for each category, which serves to evaluate the specific learning state of that category.

4 $$\begin{aligned} {{T}_{c}}(t)=\left\{ \begin{array}{*{35}{l}} \frac{1}{{{N}_{c}}}, &{} \text { if }t=0 \\ \lambda {{T}_{c}}(t-1)+(1-\lambda )\frac{1}{B}\sum \limits _{b=1}^{B}{\max }\left( p_{i}^{u}(c) \right) , &{} \text { otherwise } \\ \end{array} \right. \end{aligned}$$

From this, we obtain the self-feedback threshold for each category as ${{T}_{c}}=[{{T}_{c}}(1),{{T}_{c}}(2),\cdots ,{{T}_{c}}({{N}_{c}})]$.

By employing both global self-feedback and intra-class self-feedback threshold learning, we can obtain the final self-feedback threshold $T_g(c)$.

5 $$\begin{aligned} T_g(c)={\text {MaxNorm}}\left( T_c(t)\right) \cdot T_g=\frac{T_c(t)}{\max \left\{ T_c(t): c \in [N_c]\right\} } \cdot T_g \end{aligned}$$

The calculation form of MaxNorm is denoted as ${x}'=\frac{x}{\max (x)}$. Based on the self-feedback threshold calculated above, a more reliable set of unlabeled samples, denoted as $\hat{X}_u$, can be obtained using the method described in Eq. 2.

#### Class-imbalance focal learning

To alleviate the impact of class imbalance in training data after sample screening on model training, this paper introduces Focal Loss [31] to perform weighted feature learning on training samples. This approach directs the model’s attention towards boundary samples, enhancing the model’s ability to learn from imbalanced classes and difficult samples. The class imbalance focal learning is defined by,

6 $$\begin{aligned} {{\mathcal {L}}_{f}}=-\mathbb {E}_{x^u_i\in \hat{X}_u}{{(1-p_{i}^{u})}^{\gamma }}\log (p_{i}^{u}) \end{aligned}$$

where $\gamma$ is set to 2.

By introducing Focal Loss, we can effectively tackle the issue of imbalance in the number of different categories, reducing the impact of the self-feedback threshold learning mechanism on the number of screened samples. Ultimately, the loss function of the model proposed in this paper is defined as follows,

7 $$\begin{aligned} \mathcal {L}={{\lambda }_{1}}{{\mathcal {L}}_{ce}}+{{\lambda }_{2}}{{\mathcal {L}}_{T}}+{{\lambda }_{3}}{{\mathcal {L}}_{f}} \end{aligned}$$

In this formulation, $\lambda _1$, $\lambda _2$ and $\lambda _3$ represent the balancing coefficients for each term in the loss function.

As described, the STFL network achieves the semi-supervised skin lesion classification task, and the pseudo algorithm is summarized in Algorithm 1.

**Algorithm 1:**
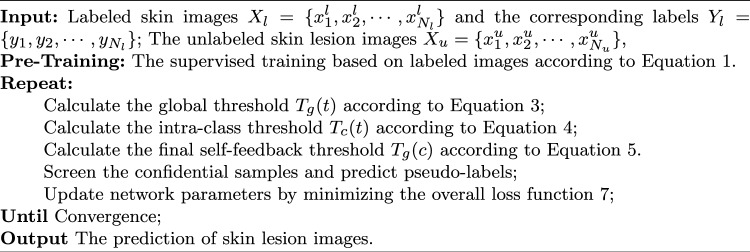
The Algorithm of STFL Network

## Experiments

### Data source of skin lesion images

The experiments in this paper are conducted on the public skin cancer diagnosis dataset HAM10000 [32]. This dataset has been collected over 20 years from two different locations: the Department of Dermatology at the Medical University of Vienna, Austria, and the Cliff Rosendahl’s Skin Cancer Clinic in Queensland, Australia. The Australian clinic uses PowerPoint files and Excel databases to store images and metadata. The Austrian site began collecting images before the digital camera era and stored images and metadata in different formats at different times, capturing dermatoscopic images from diverse populations.

As the largest public dataset of skin lesion images currently available, it contains 10,015 images of 7 types of skin lesions, including actinic keratosis and intraepithelial carcinoma/Bowen’s disease AKIEC (327 images), basal cell carcinoma BCC (514 images), melanoma MEL (1113 images), benign keratosis-like lesions BKL (1099 images), dermatofibroma DF (115 images), melanocytic nevus NV (6705 images), and vascular lesions VASC (142 images). The image quantity and description is summarized in Table 1. More than half of the subjects are diagnosed through histopathology, while the remaining patients are confirmed through follow-up examinations, expert consensus, or in vivo confocal microscopy. In our semi-supervised self-feedback focal learning model, this dataset is randomly divided into a training set of 5000 images and a test set of 5015 images. The training set is further divided into labeled and unlabeled subsets. To closely mimic the diverse scale of labeled data often encountered in clinical settings and to validate the effectiveness of our model across a spectrum of real-world scenarios, our experiments were designed to include a variety of labeled data subsets, specifically 500, 1000, 1500, 2000, 2500 and full labeled samples. This stratified approach allows us to thoroughly assess the adaptability and diagnostic accuracy of our semi-supervised model under the fluctuating availability of expert-annotated images, as is commonly the case in medical practice.

**Table 1:** HAM10000 Skin lesion image quantity and description

Abbreviation	Lesion type	Image count	Brief description
AKIEC	Actinic keratosis and intraepithelial carcinoma/Bowen’s disease	327	A form of early skin squamous cell carcinoma, typically appearing on sun-exposed skin
BCC	Basal cell carcinoma	514	The most common type of skin cancer, slow-growing, often on the head and neck
MEL	Melanoma	1,113	A severe form of skin cancer, known for irregular pigmented lesion changes
BKL	Benign keratosis-like lesions	1,099	Common benign lesions including seborrheic keratosis and solar lentigo
DF	Dermatofibroma	115	Common benign skin lesions, appearing as small hard nodules or spots
NV	Melanocytic nevus	6,705	Generally benign moles, extensively present on the skin
VASC	Vascular lesions	142	Often involve blood vessels, like hemangiomas, manifesting as prominent red spots on the skin

### Experimental settings

In the experimental process of this paper, we adopt ResNet50 as the feature extractor, initialize it with the model parameters pre-trained on ImageNet [33], and train for 150 epochs using the Stochastic Gradient Descent (SGD) optimizer [34]. The batch size is set to 64, and the initial learning rate is 0.001. All images are resized to $224 \times 224$ pixels, and data augmentation is applied using random horizontal flipping. The model experiment is implemented in Pytorch on an NVIDIA Geforce 4090 GPU. As for parameter setting, $\lambda _{1}$, $\lambda _{2}$, and $\lambda _{3}$ are set to 1, 0.5 and 0.5 respectively. Furthermore, to evaluate the performance of the model, this paper uses accuracy, precision, sensitivity, specificity, the Kappa coefficient, and AUC values as evaluation metrics for model assessment. Detailed introductions of these metrics are as follows:

(a) Accuracy, as the most intuitive performance evaluation metric, accuracy represents the proportion of correct predictions made by the model. The formula for calculating accuracy is as follows:

8 $$\begin{aligned} \text {Accuracy} = \frac{\text {TP} + \text {TN}}{\text {TP} + \text {FP} + \text {FN} + \text {TN}} \end{aligned}$$

Here, TP stands for True Positive, TN for True Negative, FP for False Positive, and FN for False Negative.

(b) Precision, refers to the proportion of actually positive samples among all predicted positive samples. The formula for calculating precision is as follows:

9 $$\begin{aligned} \text {Precision} = \frac{\text {TP}}{\text {TP} + \text {FP}} \end{aligned}$$

(c) Sensitivity (also known as True Positive Rate or Recall), refers to the proportion of samples that are actually positive and are correctly identified as positive. The formula for calculating sensitivity is as follows:

10 $$\begin{aligned} \text {Sensitivity} = \frac{\text {TP}}{\text {TP} + \text {FN}} \end{aligned}$$

(d) Specificity, refers to the proportion of samples that are actually negative and are correctly identified as negative. The formula for calculating specificity is as follows:

11 $$\begin{aligned} \text {Specificity} = \frac{\text {TN}}{\text {TN} + \text {FP}} \end{aligned}$$

(e) The Kappa coefficient $\kappa$ is a statistical measure of inter-rater agreement or accuracy, taking into account the possibility of agreement occurring by chance. It is calculated using the formula,

12 $$\begin{aligned} \kappa = \frac{p_o - p_e}{1 - p_e} \end{aligned}$$

where $p_o$ is the observed agreement ratio, $p_e$ is the expected agreement by chance, computed as $p_e = \sum _{i=1}^{k} \frac{(n_{i, \cdot } \cdot n_{\cdot , i})}{n^2}$. In this equation, $n_{i, \cdot }$ is the total of row *i* (actual class, $n_{\cdot , i}$ is the total of column *i* (predicted class),*n* is the total number of observations, and *k* is the number of classes.

(f) AUC (Area Under the ROC Curve), is the area under the Receiver Operating Characteristic (ROC) curve, used to assess the performance of the classifier. The AUC value ranges between 0 and 1, with larger values indicating better classification performance.

### Experimental performance

#### Result analysis

To validate the effectiveness of the skin cancer diagnosis model based on semi-supervised self-feedback focal threshold learning, this study designed a series of experiments on the HAM10000 dataset, and made a detailed analysis of the experimental results with different numbers of labeled samples (500, 1000, 1500, 2000, 2500, and full 5000). Notably, with only 500 labeled samples, the model has already demonstrated quite encouraging performance. As shown in Table 1, the model achieves an accuracy of 0.77 under the condition of 500 labeled samples, indicating even with fewer samples, the model can achieve high prediction accuracy. Moreover, the Kappa coefficient is 0.6408, displaying a moderate level of consistency; the F1 score is 0.7462, reflecting a good balance between precision and recall rate; the recall rate and precision are 0.77 and 0.7426, respectively, verifying the effectiveness of the model in identifying true positive samples.

As the number of labeled samples increases from 1000 to 5000 (fully labeled), the performance of the model shows a significant improvement. Particularly, in the case of 5000 labeled samples (i.e., the complete dataset), all the model’s metrics reach the highest values: accuracy is 0.8550, Kappa coefficient is 0.8058, F1 score is 0.8507, recall rate and precision are 0.8486 and 0.8550, respectively. These high-level metrics suggest that the model has extremely high skin lesion diagnostic performance on large-scale datasets. The significant improvements in accuracy and Kappa coefficient emphasize the model’s accuracy and consistency in diagnosing skin cancer, while the outstanding performance of the F1 score, recall rate, and precision further confirms the model’s superior performance in balancing misjudgments and missed diagnoses, highlighting its efficiency and reliability in handling complex dermoscopic image data.

**Table 2:** Experimental results of STFL with different labeled data scales

Labeled Scale	Accuracy	Kappa	Recall	Precision	F1-score
500	0.77	0.6408	0.77	0.7426	0.7462
1000	0.7937	0.7010	0.7937	0.7762	0.7784
1500	0.8085	0.7313	0.8085	0.7953	0.7979
2000	0.8189	0.7496	0.8189	0.8076	0.8097
2500	0.8315	0.7660	0.8315	0.8260	0.8267
5000	0.8550	0.8058	0.8486	0.8550	0.8507

**Fig. 3:**
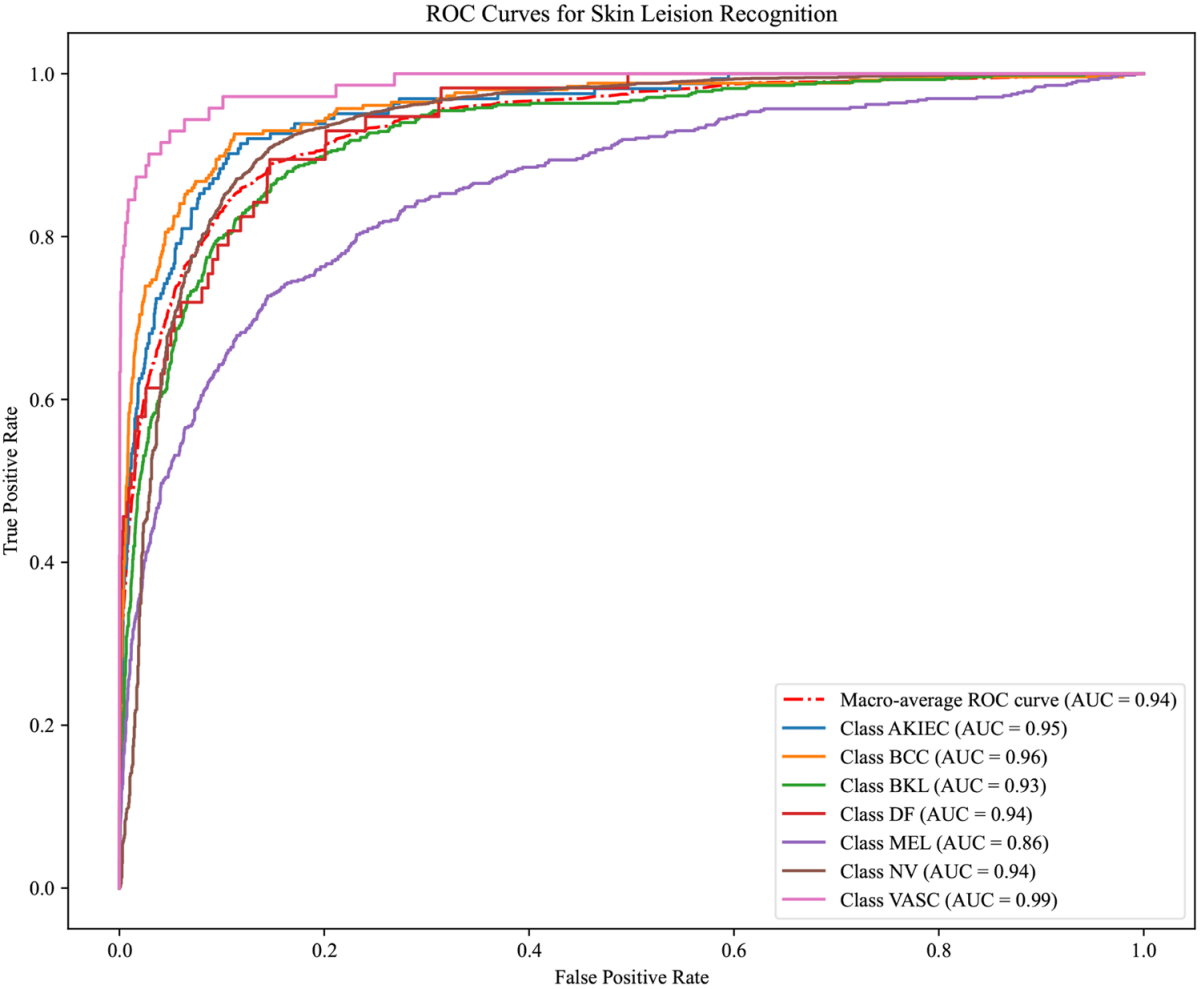
The ROC curve of each category in this model under 500 annotated samples

Besides, we adopt the Receiver Operating Characteristic (ROC) curve to conduct further result analysis in skin cancer diagnosis. As shown in Fig. 3, the ROC curve of 500 labeled samples indicates that our model achieves outstanding performance across different types of skin cancer diagnosis tasks. In identifying various types of skin lesions such as AKIEC (Actinic Keratoses and Intraepithelial Carcinoma), BCC (Basal Cell Carcinoma), BKL (Benign Keratosis-like Lesions), DF (Dermatofibroma), MEL (Melanoma), NV (Melanocytic Nevi), and VASC (Vascular Lesions), our model attains AUC values of 0.95, 0.96, 0.93, 0.94, 0.86, 0.94 and 0.99, respectively, with particularly prominent diagnostic performances for BCC and AKIEC. At the same time, a AUC of macro-average ROC curve of 0.94 further substantiates the robustness of our model in addressing multi-class skin cancer diagnosis problems.

The experimental results in ROC curves along with the diagnostic performance in Table 2 validate the effectiveness of the semi-supervised self-feedback focal learning method in skin cancer diagnosis, and demonstrate the potential of our model to achieve high accuracy in multi-class tasks with limited labeled samples. Through an in-depth analysis of the ROC curves, the diagnostic capability of our model in distinguishing different types of skin lesions is clearly visible, which is of crucial significance for clinical applications.

**Fig. 4:**
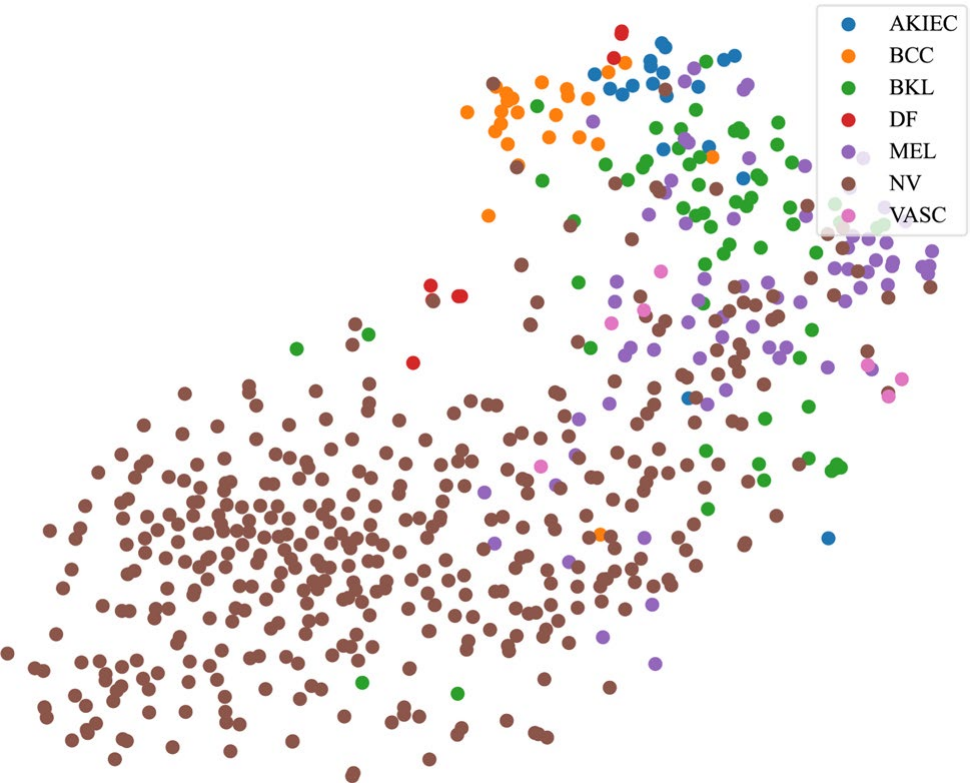
The t-SNE visualization in this model under 2500 annotated samples

Furthermore, the t-distributed Stochastic Neighbor Embedding (t-SNE) algorithm is employed to provide a visualization analysis of high-dimensional data from 2500 labeled samples produced by our model, thus intuitively displaying the separation effect of our model on different types of skin lesions in the feature space. The t-SNE is a nonlinear dimensionality reduction technique particularly suited to embedding high-dimensional datasets into a 2D or 3D space, thereby offering a visual representation of patterns and groups within the dataset. As shown in Fig. 4, the t-SNE technique maps the high-dimensional feature space of the model onto a two-dimensional plane. In this figure, each point represents a skin image sample, and different colors of markers correspond to different types of skin lesions, including AKIEC, BCC, BKL, DF, MEL, NV, and VASC. It can be observed that samples of different categories form distinct clusters in the two-dimensional space, demonstrating the ability of our model to effectively differentiate different lesion types in a high-dimensional space.

Particularly noteworthy is the relatively dispersed distribution of Basal Cell Carcinoma (BCC) and Melanoma (MEL) in the feature space, which may reflect the heterogeneity of these lesions in clinical features. Meanwhile, the samples of Melanocytic Nevi (NV) aggregate into a unique area in the figure, indicating that our model is capable of effectively distinguishing it from other types of lesions. This differentiation ability is crucial for reducing false positives during the diagnosis process, especially in the early diagnosis of skin cancer.

For the ROC analysis, we used results from 500 labeled samples to emphasize that our proposed semi-supervised learning model can achieve satisfactory classification performance even with a limited amount of labeled data. This is particularly important for clinical settings with limited data annotation resources, as we aim to demonstrate that the STFL model is practical even with fewer labeled samples; As for the t-SNE analysis, since it is a high-dimensional data visualization technique intended to show the distribution of data in a low-dimensional space, conducting t-SNE analysis on a larger dataset provides us with a clearer and more comprehensive view of the internal structure of the data. We chose 2500 labeled samples to demonstrate the stability and effectiveness of the model’s capability when dealing with larger volumes of data. Such a display helps assessors better understand the model’s generalization ability across different scales of data.

The visualization results in this section further substantiate the superior performance of our model in feature extraction and classification. The t-SNE plot provides us with an intuitive validation tool, proving that the features learned by the model have good discriminatory power for different types of skin lesions. Furthermore, this also offers important intuitive evidence for future model improvements and optimizations. By analyzing the t-SNE results, we can adjust the model structure and parameters specifically, ultimately enhancing the application performance of the model in the field of automatic skin cancer diagnosis.

### Comparison with state-of-the-arts

In this part, we provide a comprehensive comparison of our proposed Semi-Supervised Self-Feedback Threshold Focal Learning (STFL) approach with other established semi-supervised methodologies evaluated on skin cancer diagnosis. As shown in Table 3, our approach utilizes only 500 labeled samples, which is notably less than the 600 samples used by other methods. To address the need for efficient diagnostic models in environments with limited annotated data, we trained our STFL model using 500 labeled images against the literature with 600 label images. This was done to (1) verify the model’s robust performance despite fewer annotations, comparing favorably with methods requiring 600 labeled examples; (2) to demonstrate the model's ability to utilize unlabeled data effectively; and (3) to challenge the convention of needing large labeled datasets for high accuracy, showing that our model can achieve comparable, superior diagnostic performance with less labels, thereby providing a compelling alternative for medical image analysis with restricted resources.

In terms of the Area Under the Curve (AUC), which is a widely accepted performance metric in medical diagnosis models, our method achieved an impressive AUC score of 0.940. This result significantly surpasses the performance of the other compared methods such as FixMatch [35] with an AUC of 0.774, Self-train [36] with an AUC of 0.769, GLM [37] with an AUC of 0.783, NM [38] with an AUC of 0.792, Bis [39] with an AUC of 0.770, and CSDA [40] with an AUC of 0.804.

**Table 3:** Comparison with the performance of state-of-the-arts with 600 labeled samples, while our STFL only utilize 500 labeled data

Method	Labeled data	AUC
FixMatch [35]	600	0.774
Self-train [36]	600	0.769
GLM [37]	600	0.783
NM [38]	600	0.792
Bis [39]	600	0.770
CSDA [40]	600	0.804
Ours	500	**0**.**940**

This comparative analysis clearly suggests that our semi-supervised STFL approach is highly effective and efficient, providing superior performance even with reduced labeled data. This promising results of our method not only push the technology frontier in semi-supervised skin cancer diagnosis but also imply its significant clinical value. In particular, the decreased reliance on labeled data reduces the manual annotation burden, which is often a limiting factor in medical AI applications. Therefore, the superiority of our method over state-of-the-art approaches signifies a substantial advancement in the field of semi-supervised learning for skin cancer diagnosis.

**Fig. 5:**
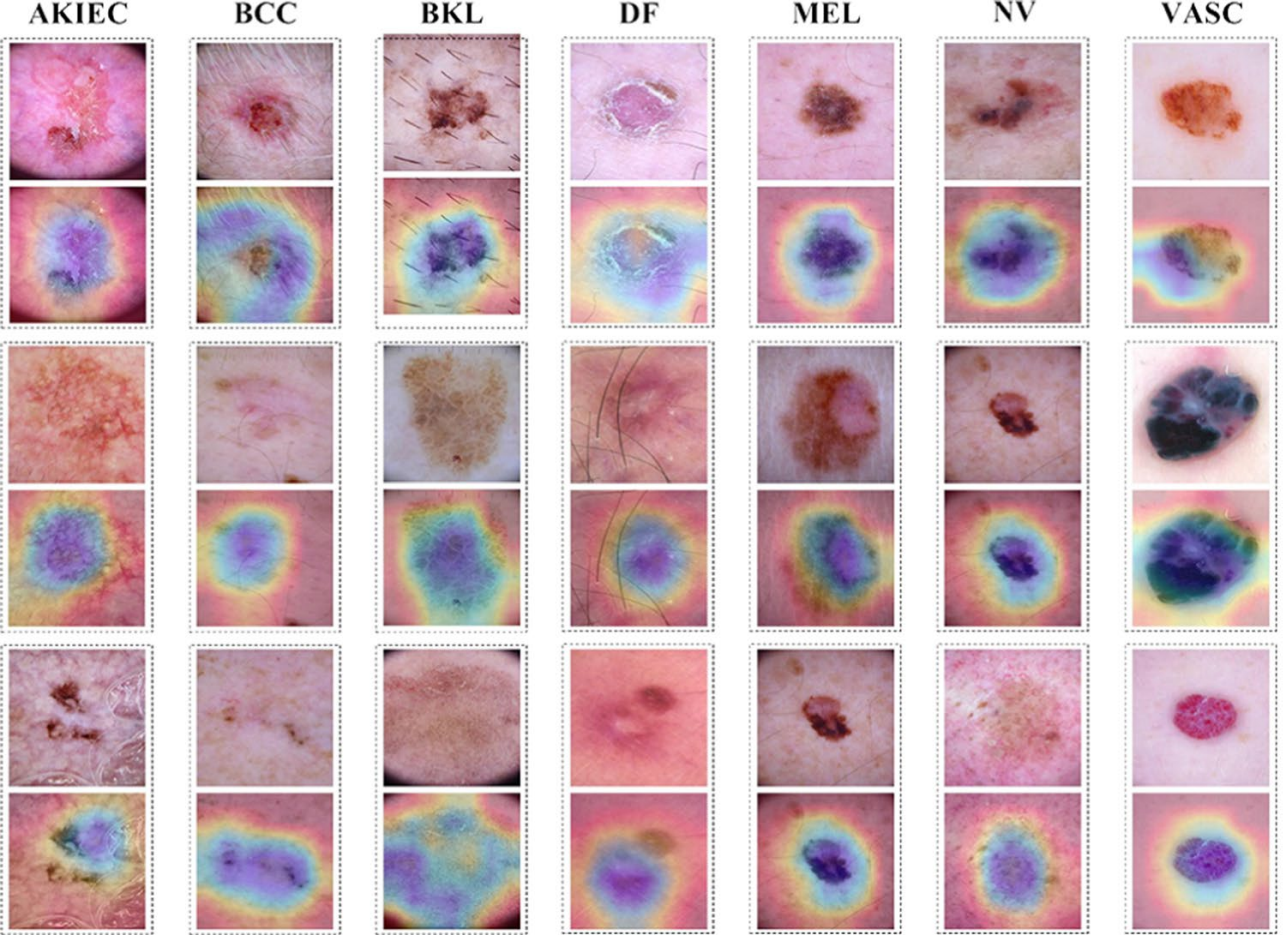
The grad-cam visualization of STFL with 2500 annotated samples. The upper is original images, and the image below is the heatmap

*Grad-CAM on Skin Images* This part delves into into the visual interpretation of our model’s decision-making process, leveraging the Gradient-weighted Class Activation Mapping (Grad-CAM) technique [41]. The Grad-CAM technique generates visual explanations for decisions from Convolutional Neural Networks (CNN)-based models, such as our STFL model, providing insights into what regions in the input image the model found most significant for its skin lesion diagnosis.

Fig. 5 illustrates the Grad-CAM visualizations for the seven types of skin lesions diagnosed by our model. The figure depicts that our model focuses on the correct regions of interest and demonstrates an impressive capability to identify and distinguish between distinct skin lesion types (consistent with professional doctors), even when trained with only 2500 labeled samples. This confirms the reliability and robustness of our model in skin cancer diagnosis. To further illustrate, the heatmaps overlaying the original images highlight the discriminative regions identified by our model. In each subfigure, the cooler colors signify the areas that have contributed most to the model’s prediction, often corresponding to the pathological characteristics of each class of lesions. For instance, in the case of Melanocytic nevi, the model has accurately fixated on the symmetrical shape and even coloration, which are key diagnostic features.

This visual evidence underlines the model’s ability to not just make accurate predictions, but also base its decisions on clinically significant features, which aligns with the diagnostic reasoning of dermatologists. Therefore, in addition to superior performance, our STFL model also offers interpretability, a crucial aspect in clinical applications to trust AI-based diagnostic tools.

### Ablation study

#### Analysis of introducing unlabeled samples in skin cancer diagnosis

To further emphasize the role of unlabeled samples in model training, this study conducted additional control experiments. As displayed in Table 4, the removal of unlabeled data led to a general decrease in model performance compared with the results shown in Table 2. Taking the scenario of 500 labeled samples as an example, the accuracy of the model decreased from 0.77 to 0.7478, the Kappa coefficient declined from 0.6408 to 0.6410, and there were corresponding decreases in the F1 score, recall, and precision. This change intuit demonstrates the significance of introducing unlabeled samples in enhancing the performance of the skin cancer diagnosis model.

**Table 4:** Experimental results of STFL without unlabeled data

Labeled Scale	Accuracy	Kappa	Recall	Precision	F1-score
500	0.7478	0.6410	0.7478	0.7478	0.6410
1000	0.7887	0.7124	0.7887	0.7887	0.7813
1500	0.7941	0.7941	0.7877	0.7838	0.7877
2000	0.8137	0.7838	0.8137	0.8067	0.8093
2500	0.8203	0.7605	0.8151	0.8120	0.8151

By effectively utilizing the information contained in unlabeled samples, our model significantly improves the accuracy and reliability of skin cancer diagnosis. This finding not only emphasizes the advantages of semi-supervised learning in leveraging limited labeled data but also offers new perspectives and methodologies for subsequent medical image processing research, providing valuable scientific evidence for the early diagnosis and treatment of skin cancer.

#### Analysis of the backbone network

In this part, we delve into the impact of different backbone networks on our proposed semi-supervised skin cancer diagnosis model, which is underpinned by Self-feedback Threshold Focal Learning (STFL). Specifically, we examine three distinct classical convolutional neural network architectures: VggNet [42], GoogleNet [43], and ResNet [27], comparing their performance with a fixed set of 500 labeled samples.

As demonstrated by the experimental results in Table 5, ResNet outperforms Vgg and GoogleNet across nearly all metrics. Notably, it achieves the highest values in accuracy, recall, precision, and F1-score, recorded at 0.77, 0.77, 0.7426, and 0.7462, respectively. These results suggest that ResNet possesses superior feature extraction capabilities and generalization performance in classifying skin lesion images. It is worth mentioning that while VggNet shows the best performance in terms of the Kappa coefficient, reaching 0.6541, this indicates a relative strength of VggNet in handling classification consistency. However, its performance in other metrics still lags behind ResNet.

**Table 5:** Experimental results of STFL with different backbone networks under 500 labeled data

Backbone	Accuracy	Kappa	Recall	Precision	F1-score
VggNet	0.737	**0**.**6541**	0.7462	0.737	0.7371
GoogleNet	0.7264	0.5730	0.6867	0.7264	0.6881
ResNet	**0**.**77**	0.6408	**0**.**77**	**0**.**7426**	**0**.**7462**

The bold value denotes the best performance

The Vgg network, known for its simplistic yet deep architecture, is hindered by its large model complexity and parameter count, which limits its efficiency and practical applicability under resource-constrained conditions. GoogleNet, with its innovative Inception module designed to capture image information at various scales, exhibits inferior results compared to ResNet and Vgg in our experiments, with an accuracy and F1-score of 0.7264 and 0.6881, respectively. This may imply that for the specific task of skin cancer diagnosis, GoogleNet’s feature extraction is less effective compared to the other two architectures.

In summary, although VggNet and GoogleNet have competitive strengths in certain metrics, ResNet, with its residual learning feature and outstanding performance, emerges as the ideal choice for employing our STFL approach in this study. These findings provide clear guidance for selecting the appropriate backbone network when constructing efficient semi-supervised skin cancer diagnosis models in the future.

## Discussion and conclusion

This study aims to address the performance inadequacies that existing supervised learning methods often exhibit when applied to new diagnostic scenarios. Traditional supervised learning models tend to experience a drastic drop in diagnostic performance when confronted with new scenarios different from the training set environment, primarily due to the lack of appropriately labeled data in the new settings. This phenomenon severely limits the generalizability of automatic diagnostic models. Hence, we face a practical challenge: how to continually optimize and enhance the adaptability of supervised learning models without relying on extensive labeling of data in new environments. The semi-supervised learning model proposed in this research, the Skin Cancer Diagnosis model based on Self-feedback Threshold Focal Learning (STFL), seeks to alleviate this issue. It aims to equip the model to more robustly handle various diagnostic settings, reduce data labeling costs, while maintaining or even improving diagnostic accuracy.

In terms of performance, the STFL model significantly surpasses traditional fully supervised learning methods, especially notable when labeled data is limited. The STFL approach enhances model performance by adaptively adjusting the selection threshold for unlabeled samples and employing focal learning to address class imbalance issues. These strategies collectively improve the model’s ability to filter uncertain samples and its generalizability in new environments. Experimental results reveal that with only 500 labeled samples, the model achieves an accuracy of 0.77, a Kappa coefficient of 0.6408, a recall of 0.77, a precision of 0.7426, and an F1-score of 0.7462, demonstrating that the STFL model can still achieve commendable accuracy even with fewer labeled samples. As the volume of labeled samples increases, the model’s performance further enhances: with 1000 labeled samples, the accuracy rises to 0.7937, with corresponding increases in Kappa coefficient, recall, precision, and F1-score. Notably, in experiments conducted with all 5000 labeled samples, the model reaches an accuracy of 0.8550, a Kappa coefficient of 0.8058, and heightened recall, precision, and F1-scores. These experimental outcomes thoroughly substantiate the STFL model’s effective utilization of a vast amount of unlabeled medical image data and its significant reduction in misdiagnosis rates without relying on excessive human intervention, offering robust scientific support for the early diagnosis and treatment of skin cancer.

In new skin cancer diagnostic scenarios, the STFL model exhibits particular advantages, demonstrating that it can effectively utilize a large amount of unlabeled data for training, even under the condition of limited annotated samples, thereby constructing a high-performance diagnostic model. This characteristic is particularly beneficial in the diverse and unevenly distributed real-world situations of skin cancer types, where certain rare or specific demographic-related skin cancer categories (such as different skin color types) may face significant data scarcity. With the STFL model, even with only a handful of labeled samples, the model can learn effective diagnostic patterns, using the vast amount of unlabeled sample data to further enhance the model’s generalization ability and diagnostic accuracy.

Moreover, the self-feedback mechanism integrated into the STFL model can dynamically adjust the selection threshold for unlabeled samples, in combination with the focal learning strategy, effectively addressing the common issue of class imbalance in medical image diagnosis. This means that the model is capable not only of identifying common types of skin cancer but also of enhancing sensitivity to rare cancer types, thereby improving the coverage and accuracy of diagnosis. In medical environments where resources are limited or obtaining a large number of annotated samples is difficult, the STFL model’s feature can provide healthcare professionals with a powerful tool, ensuring that all types of skin cancer patients can receive timely and accurate diagnoses. This not only reduces the workload and cost of data annotation but also increases the efficiency of diagnosis, while also enhancing the accessibility and feasibility of the diagnostic process. Therefore, the STFL model not only provides a new perspective for skin cancer diagnosis in new scenarios but also offers an effective solution to the key challenges in medical image analysis, such as data scarcity and class imbalance.

In practice, our STFL model is not confined to the diagnosis of skin cancer; its principles and methods can also be extended to other diagnostic imaging analyses, such as the analysis of pathological slides or the early detection of other types of cancer. Through semi-supervised learning, the reliance on labeled data can be greatly reduced. This holds transformative potential especially for the medical field where data is scarce, particularly in regions lacking in expertise or technology.

*Industrial significance* The Self-feedback Threshold Focal Learning (STFL) model introduces an important industrial advance in the field of semi-supervised skin cancer diagnosis, offering a cost-effective and scalable solution for healthcare providers. By reducing reliance on extensive labeled datasets, it lowers operational costs and makes high-quality diagnostic services more accessible, especially in underserved areas. The model enhances clinical workflow efficiency by automating preliminary analysis, allowing clinicians to concentrate on complex cases. Furthermore, its self-adaptive capabilities ensure it remains effective as new data becomes available, mitigating the risk of misdiagnosis associated with class imbalance. The potential of STFL extends beyond skin cancer, promising applications in various domains of diagnostic medicine. This represents a substantial step towards more efficient, effective, and accessible medical diagnostics, highlighting its significant value to the healthcare industry.

*Limitations of the Study* Despite the impressive performance of the STFL model in our experiments, there are limitations. The performance of the model is bounded by the quality and quantity of unlabeled data, where inaccurate or poor-quality unlabeled data could adversely affect the learning process and outcomes. Furthermore, the model may not perform as well on specific types of skin cancer or on image datasets of certain ethnicities, necessitating further validation and enhancement of its universal adaptability.

*Future Works* We will focus on further enhancing the generalization capabilities of the STFL model, including tests for adaptability across different skin types and a range of poor-quality unlabeled data. Improvements in detection performance and model adaptability, as well as integration into actual clinical workflows, are among the priorities for future work. We also plan to explore the model’s application in real-time diagnostic environments and assess its potential impact on improving patient treatment outcomes. Finally, bridging the gap between manual annotation of data and machine learning models, to achieve more precise and efficient medical diagnoses, remains an unwavering goal of our future efforts.

## Data Availability

The datasets generated during and/or analysed during the current study are available from the corresponding author on reasonable request.
